# 2021 Annual Meeting of the Swiss Society for Sleep Research, Sleep Medicine, and Chronobiology (SSSSC)

**DOI:** 10.3390/clockssleep4020026

**Published:** 2022-06-07

**Authors:** Martin Hatzinger

**Affiliations:** SSSSC President, Psychiatric Services, Solothurner Spitäler AG, Solothurn and Medical Faculty, University of Basel, Weissensteinstrasse 102, CH-4503 Solothurn, Switzerland; martin.hatzinger@spital.so.ch

The 2021 meeting in Solothurn provided evidence-based education to advance the science and clinical practice of sleep medicine and sleep physiology, disseminates cutting-edge sleep and circadian research, promotes the translation of basic science into clinical practice, and fosters the future of the field by allowing young clinicians and researchers to present their findings in talks and on posters. Renowned international and national speakers provided comprehensive updates, educational workshops, and insights into novel scientific topics covering various areas of sleep research and sleep medicine. In addition, a workshop of psychotherapy in insomnia was provided by the “Special Interest Group (SIG) Psychiatry and Sleep.” This course was suitable to earn the credits necessary for the certificate of capacity in sleep medicine, especially for non-psychiatrists. Most importantly, the meeting provided ample opportunities to bring together people, to engage in discussions, and to plan scientific projects.

## 1. Content

**Table:** 

Basic Science			
No.	Title	Author	Page
1	Does Auditory Slow Wave Stimulation Affect Motor Fatigability?	Manuel Carro Dominguez	5
2	Pre-Sleep Intentions to React to Stimuli during Sleep Impair Sleep and Alter Stimulus Processing during Sleep	Selina Ladina Combertaldi	6
3	Subjective Sleep Quality Is Reduced in People Declaring Themselves as Electrohypersensitive	Corinne Eicher	7
4	Local Manipulation of Sleep Depth by Auditory Stimulation during the Down Phase of Slow Waves	Sara Fattinger	8
5	Automatized Selective Slow-Wave Sleep Suppression	Kristoffer D. Fehér	9
6	Effects of Auditory Stimulation Modalities on Cardiovascular Dynamics during Sleep	Stephanie Huwiler	10
7	Proof-of-Concept Study Using Phase-Targeted Auditory Stimulation during Sleep to Boost Slow-Wave Activity in Children with Attention-Deficit Hyperactivity Disorder (ADHD)	Elena Krugliakova	11
8	Measuring Regulation of Pupil Size Under Real-World Conditions	Rafael Lazar	12
9	Are Interictal Epileptiform Discharges during Sleep Causally Related to Slow Waves? Investigating their Relationship Using Down-Phase-Targeted Auditory Stimulation: A Pilot Study	Sven Leach	13
10	Spectral Slope between 30–45 Hz during NREM and REM Sleep Differentiates between Insomnia Patients and Healthy Controls	Christian Mikutta	14
11	Circadian VIPergic Neurons of the Suprachiasmatic Nuclei Directly Control Siesta Sleep	Sara Pierre-Ferrer	15
12	Does Enhancing Slow-Wave Activity Using Auditory Stimulation Boost the Restorative Function of Sleep?	Niklas Schneider	16
13	Effects of Sleep Deprivation on Task EEG Theta Power	Sophia Snipes	19
14	A Sustained and Vicious Cycle? Bidirectional Relationships between Sleep and Stress in Depressed and Healthy Adolescents	Salome Wild	20
15	Physiological Responsiveness to Phase-Locked Auditory Stimulation during SWS Predicts Increases in Episodic Memory Performance in Older Adults	Marina Wunderlin	22
16	Improving Sleep to Prevent Cognitive Decline	Céline Zeller	23
**“Clinical Science”**			
17	Sleep Neurophysiology in Unmedicated Adolescents with and without Depression	Chiara Fontanellaz-Castiglione	27
18	More than Just Legs: Periodic Chin Muscle Activations in a Case of Abdominal RLS	Stephany Fulda	29
19	Cognitive Behavioral Therapy for Insomnia in Patients with Mental Disorders and Comorbid Insomnia: A Systematic Review and Meta-Analysis	Elisabeth Hertenstein	32
20	Heart Rate Variability in Non-Rapid Eye Movement Sleep Stage 2 Indicates Insomnia and Is Related to Subjective Daytime Performance	Thorsten Mikoteit	33
21	Sleep Alterations in Unmedicated Patients with Major Depressive Disorder Compared to Healthy Controls	Ximena Omlin	34
22	The Life-ON Study: Sleep and Sleep Disorders During Pregnancy and Postpartum	Silvia Riccardi	36
23	Obstructive and Central Sleep Apnea in First Ever Ischemic Stroke Are Associated with Different Time Course and Autonomic Activation	Alessia Riglietti	37
24	Become Your Own SLEEPexpert: Development and Evaluation of a Web Application to Support a Pragmatic Behavioral Treatment Program for Insomnia in In-Patient Psychiatric Care	Carlotta Schneider	38
25	Restless Legs Syndrome and Polysomnographic Features in Patients with Multiple Sclerosis	Davide Sparasci	39
26	Cognitive Behavioral Therapy for Insomnia in Patients with a Psychiatric Comorbidity: A Systematic Review and Meta-Analysis Focusing on Comorbid Symptoms	Ersilia Trinca	43

## 2. Basic Science

### 2.1. Does Auditory Slow Wave Stimulation Affect Motor Fatigability?

Carro Dominguez, M ^1^, Huwiler, S ^1^, Stich, F ^1^, Aziri, F ^1^, Sala, R ^1^, Trippel, A ^1^, Huber, R ^2^, Schmied, C ^3^, Meissner, S ^1^, Wenderoth, N ^1^ and Lustenberger C ^1^

^1^ Department of Health Sciences and Technology, ETH Zurich, Zurich, Switzerland

^2^ University Children’s Hospital Zurich, University of Zurich, Zurich, Switzerland

^3^ University Hospital of Zurich, Zurich, Switzerland

**Background:** Slow waves (SWs) during sleep are proposed to be central in restoring different brain and body functions. Yet, little is known about the restorative function of sleep in motor fatigability—a gradual decrease in performance when a motor task is executed over an extended period. Supraspinal mechanisms have been shown to play an essential role in evoking this decrease in performance. Such mechanisms may be further understood by elucidating the influence of locus coeruleus norepinephrine system dynamics, which can be indirectly monitored with pupillometry, the recording of pupil size. Finger tapping is a motor task where an increase in inter-tap intervals (ITI) reflects motor fatigability. To investigate whether SWs influence motor circuit resistance against fatigability, we applied auditory stimulation known to enhance SWs and established the effect on overnight changes in finger tapping performance. **Methods**: 18 healthy, middle-aged (30–57 years of age) male participants underwent 3 nights in the sleep lab where one of three auditory stimulation (stim) modalities was applied in a counter-balanced, cross-over design (no stim, low-volume stim, high-volume stim). Finger tapping was tested while recording pupil size in the evening before and the morning after each sleep period. Lighting conditions were kept constant throughout the task. **Results:** Linear mixed-effects modeling revealed lower ITI in the morning (*p* < 0.05), particularly in late stages of the tapping period (*p* < 0.01). ITI variability was greater in the morning (*p* < 0.01), particularly in the high-volume stim condition (*p* < 0.05). Pre-tapping pupil size was lower in the morning (*p* < 0.01) and negatively correlated with the size of maximum pupil dilation during initial tapping (r(579) = −0.32, *p* < 0.001). **Conclusions**: These findings suggest that sleep might modulate motor circuit resistance against fatigability and that auditory slow-wave stimulation influences variability in motor performance Overnight differences in pupil dynamics are evident, but their relationship with motor performance remains to be analyzed.

**Funding:** This work was conducted as part of the SleepLoop Flagship of Hochschulmedizin Zürich and funded by the Swiss National Science Foundation (PZ00P3_179795 to CL).

### 2.2. Pre-Sleep Intentions to React to Stimuli during Sleep Impair Sleep and Alter Stimulus Processing during Sleep

Selina Ladina Combertaldi, Anna Zoé Wick and Björn Rasch

Department of Psychology, University of Fribourg, Fribourg, FR, Switzerland

Sleep is a state of strongly reduced consciousness. Still, pre-sleep intentions to react to specific stimuli during sleep can strongly affect the sleep process. For example, being on call as a firefighter or medical doctor will facilitate reactions to waking sounds during sleep. However, the exact contributions of the pre-sleep intention itself on the sleep process is still not fully understood. Here we replicated a study by Wuyts et al. (2012) and tested the impact of pre-sleep intentions to react to stimuli independently from the actual sound presentation during sleep. In addition to the manipulation of the original study, we introduced a “sound” group, where sounds were actually presented. Twenty-six healthy young participants spent two nights in the sleep laboratory. Sleep was recorded using polysomnography. In one night, they were instructed to react to sounds during sleep (“on call” condition), but did not need to react to sounds the other night (“neutral” condition), in a balanced order. Unknown to the subjects, sounds were only presented in one group during both nights (“sound” group), whereas no sounds were presented in any of the two nights in the other group (“no sound” group). In accordance with our hypotheses, the instruction of being “on call” decreased objective sleep efficiency similarly in both groups, independently of sounds being present during sleep. In addition, in the “sound” group, event-related potentials in response to sounds as well as slow-wave activity were reduced in the “on call” condition compared with the “neutral” condition. Our results show that pre-sleep intentions to react to stimuli impairs sleep independently of sounds actually being present. Furthermore, pre-sleep intention alters the processing of sounds during sleep. Our results enhance our understanding of the impact of pre-sleep cognitive activity on sleep processes. In addition, they highlight the importance of subjective relevance for reducing the negative impact of external noise sources like traffic or church bells, thereby improving sleep.

### 2.3. Subjective Sleep Quality Is Reduced in People Declaring Themselves as Electrohypersensitive

Corinne Eicher ^1^, Benjamin Marty ^1^, Peter Achermann ^2,3,4,5^, Reto Huber ^2,3,4,6,7^ and Hans-Peter Landolt ^1,2,3,4^

^1^ Institute of Pharmacology and Toxicology, University of Zürich, Zürich, Switzerland

^2^ Sleep & Health Zürich, University Center of Competence, University of Zürich, Zürich, Switzerland

^3^ Neuroscience Center Zurich, University of Zurich and ETH Zurich, Zurich, Switzerland

^4^ Center for Integrative Human Physiology, University of Zurich, Zurich, Switzerland

^5^ The KEY Institute for Brain-Mind Research, Department of Psychiatry, Psychotherapy and Psychosomatics, University Hospital of Psychiatry Zurich, Zurich, Switzerland

^6^ Department of Child and Adolescent Psychiatry and Psychotherapy, Psychiatric University Hospital Zurich, Zurich, Switzerland

^7^ Child Development Center, University Children’s Hospital Zurich, Zurich, Switzerland

**Introduction**: Controlled laboratory studies demonstrate consistent effects of standardized radio frequency electromagnetic fields (EMF) on sleep EEG, yet their health significance is unclear. Disturbed sleep is a frequent complaint of people considering themselves EMF hypersensitive (EHS). Single-nucleotide polymorphisms (SNPs) in the L-type calcium channel subunit gene *CACNA1C* were previously associated with self-rated sleep quality, and the encoding protein CACNA1C may contribute to the molecular mechanisms mediating EMF effects on sleep. We investigated whether subjective sleep quality is associated with EHS and whether genetic variation in *CACNA1C* contributes to individual differences in sleep quality and EHS. **Methods**: A total of 2040 participants (1381 females) aged 18–30 years completed the Pittsburgh Sleep Quality Index (PSQI), Epworth Sleepiness Scale, Munich Chronotype Questionnaire, and a validated questionnaire on EMF sensitivity online. They also provided a saliva sample to genotype two functional SNPs of *CACNA1C*. Participants endorsing the question “Are you electro-hypersensitive?” were considered EHS, those believing to develop detrimental health symptoms due to prevailing electromagnetic pollution were considered “attributers,” and the remaining participants were considered “non-EHS.” The EHS group (*n* = 125) and the attributers (*n* = 299) were combined for binomial analyses. Associations between EMF sensitivity, subjective sleep variables, and genotype were tested by general linear models with age, gender, and habitual mobile phone use as covariates. **Results**: Compared to non-EHS, being EHS or an attributer was associated with reduced subjective sleep quality (mean ± standard deviation: PSQI 6.1 ± 2.4 vs. 5.1 ± 2.1, *p* = 1.70 × 10^−12^) and prolonged sleep latency (28.7 ± 30.0 min vs. 23.7 ± 20.6 min, *p* = 0.0021) on the PSQI, whereas mobile phone use did not affect these variables (*p* > 0.1). No associations were found between the *CACNA1C* genotype and the tested subjective sleep quality variable or EMF sensitivity status. **Conclusions**: EHS individuals reported reduced subjective sleep quality, irrespective of reported EMF exposure. Previous associations of smaller genome-wide association studies between *CACNA1C* genotypes and sleep quality could not be replicated in the current sample.

**Funding:** The study was supported by the Swiss Federal Office of the Environment.

### 2.4. Local Manipulation of Sleep Depth by Auditory Stimulation during the Down Phase of Slow Waves

Sara Fattinger ^1,2^, Georgia Sousouri ^1,2^, Carina Volk ^1,2^, Natalie Heyse ^1,2^, Fatime Bislimi ^1,2^, Valeria Jaramillo ^1,2^, Toon de Beukelaar ^3^, Kathy Ruddy ^4^, Nicole Wenderoth ^3,4^ and Reto Huber ^1,2,5^

^1^ Children’s Research Center, University Children’s Hospital Zurich, Zurich, Switzerland

^2^ Child Development Center, University Children’s Hospital Zurich, Zurich, Switzerland

^3^ Movement Control and Neuroplasticity Research Group, Department of Kinesiology, KU Leuven, Leuven, Belgium

^4^ Neural Control of Movement Lab., Department of Health Sciences and Technology, ETH Zurich, Zurich, Switzerland

^5^ Department of Child and Adolescent Psychiatry and Psychotherapy, Psychiatric Hospital, University of Zürich, Zurich, Switzerland

**Background**: EEG slow waves (SW), the intrinsic brain oscillations of deep sleep, have become a primary target of closed-loop auditory stimulation to modulate brain activity during sleep. By applying such stimulation targeting the down-phase of SW, we showed a region-specific perturbation of SW and its recovery processes, whereas global sleep and the natural sleep pattern were preserved. Here we aim at investigating key parameters in the EEG that may determine the success of such local SW modulation. **Methods**: In a retrospective analysis, data from 4 different studies targeting 4 different brain areas by closed-loop auditory down-phase stimulation were considered. In 42 subjects (22.3 ± 0.4 years old) all-night EEGs were recorded twice one night with (STIM) and one night without (NOSTIM) auditory stimulation. During STIM nights, tones (50 ms duration, ~50 dB) were delivered time-locked to the down-phase of slow waves detected in real time over the targeted area. **Results**: In three out of the four studies a focal reduction of SWA of ~12.5 ± 2.3% close to the target area in a cluster of ~ 7.3 ± 1.2 electrodes was observed. Our combined, comparative analyses showed that the following three parameters may be key for such local SWA reduction: (1) the number of targeted waves, (2) the phase-timing, and (3) the slope of the targeted wave. Thus, more specifically, we found that the simulation was most effective if tones were presented precisely after the onset of the EEG ascending trend on steep slow waves. **Conclusions**: Closed-loop stimulation to modify ongoing oscillatory brain activity may open up new treatment approaches in disorders with altered brain activity. Thus, our results represent a first attempt at enabling a disorder-specific, personalized stimulation approach, which is key for long-term, patient-oriented studies.

### 2.5. Automatized Selective Slow-Wave Sleep Suppression

Kristoffer D. Fehér ^1^, Ximena Omlin ^1^, Carlotta L. Schneider ^1^, Leila Tarokh ^1^, Yosuke Morishima ^1^, Thomas Koenig ^1^, Elisabeth Hertenstein ^1^, Ersilia Trinka ^1^, Benjamin Ellenberger ^2^, Simon Ruch ^2^, Marc A. Züst ^3^, Christian Mikutta ^1,4^ and Christoph Nissen ^1^

^1^ University Hospital of Psychiatry and Psychotherapy, University of Bern, Bern, Switzerland

^2^ Cognitive Neuroscience of Memory and Consciousness, Institute of Psychology, University of Bern, Bern, Switzerland

^3^ University Hospital of Old Age Psychiatry and Psychotherapy, University of Bern, Bern, Switzerland

^4^ Privatklinik Meiringen, Meiringen, Switzerland

**Background**: Recent studies indicate that selective suppression of slow-wave sleep (SWS), potentially through modifications of synaptic plasticity, may represent an alternative to therapeutic sleep deprivation in patients with major depression. The purpose of this project was to develop a fully automatized selective suppression of SWS based on closed-loop auditory stimulation. **Methods**: Two new automatized SWS suppression protocols using continuous and pulsed noise stimulation were developed and evaluated in a sleep laboratory study in a healthy young population (N = 10 and N = 15, respectively). Stimulation was applied upon detection of SWS. SWS detection relied on a topographical template of slow waves. For the continuous noise protocol, stimulation involved brown noise increasing from 0 to 88 dB in 300 s, until SWS was no longer detected by the algorithm. For the pulsed-burst protocol, stimulation involved applying bursts of pink noise with a randomized duration of 50–500 ms. The volume increased from 40 dB to 106 dB in 60 s, until SWS was no longer detected by the algorithm. The linear increase in volume was combined with random walks between +−2.5 dB (Ornstein–Uhlenbeck process) to add unpredictability in volume. The inter-stimulus interval was randomized between 1 and 4 s. **Results**: Contrary to our prediction, the continuous noise protocol resulted in a significant increase in SWS throughout the night, as well as a reduction in transition probability from SWS to N2, and vice versa. Slow-wave activity averaged across the night and cumulative slow-wave energy at the end of the night were both increased by about 25% and 30%, respectively, across channels and individuals. Theta power averaged across the night was increased by about 15% across channels and individuals. SW duration during SWS was decreased by about 5%, mainly in frontal channels. In line with our prediction, the pulsed-burst protocol led to a significant reduction in SWS, with an associated increase in sleep stage N2, without other significant changes in sleep continuity or architecture throughout the night. However, in the first two hours after sleep onset, the stimulation also caused an increased arousal index and wakefulness. Slow-wave activity averaged across the night and cumulative slow-wave energy at the end of the night were both reduced by about 30% across channels and individuals, without changes in other frequency bands. SW count during SWS was reduced by about 50% across channels, and SW amplitude during SWS was reduced by about 18% in fronto-central channels. **Conclusions**: We demonstrate, to our knowledge for the first time, that a fully automatized closed-loop approach using pulsed bursts of noise can suppress SWS selectively. We also demonstrate that continuous closed-loop application of noise during SWS can serve to selectively deepen SWS. Further developments bear the potential for translation to broader and even ambulatory use of automated SWS detection and modulation and related processes of brain function and health.

**Funding:** The project was funded through “IRC Decoding sleep: from neurons to health and mind,” University of Bern.

### 2.6. Effects of Auditory Stimulation Modalities on Cardiovascular Dynamics during Sleep

Huwiler, S, Huwyler, S, Kiener, L, Wenderoth, N and Lustenberger, C

Department of Health Sciences and Technology, ETH Zurich, Zurich, Switzerland

**Background**: Deep non-rapid eye movement (NREM) sleep is considered an important period of restoration for the brain and the body. During this period, the parasympathetic branch of the autonomic nervous system, which is the main link between the brain and the cardiovascular system, predominates over the sympathetic nervous system. Yet, functionally linking brain oscillations during deep NREM sleep and their effects on cardiovascular dynamics has been neglected. **Methods**: To elucidate the role of slow-waves hallmarking deep NREM sleep in cardiovascular dynamics, we applied various auditory stimulation modalities (targeting the up-phase of slow waves, targeting the down-phase of slow waves, and 1 Hz rhythmic stimulation (ISI1)) and a sham stimulation, which were all presented within a single nocturnal period in a windowed ON–OFF approach. A total of 23 healthy male participants (age 19–59) underwent one night of polysomnographic recordings using high-density EEG and simultaneous ECG recordings. **Results**: We found mean heart rate to be significantly increased in the ON windows of down-phase and ISI1 stimulation compared to no stimulation (sham). Furthermore, mean heart rate was significantly decreased for the up-phase, down-phase, and ISI1 in the OFF window compared to no sham stimulation (all *p* < 0.05). Moreover, we found RMSSD, a heart-rate variability measurement indirectly estimating parasympathetic activity, to be increased in the ON window for down-phase stimulation (*p* = 0.03) compared to sham stimulation. **Conclusions**: Overall, our findings showed that auditory slow-wave modulation evokes a first acceleration followed by a deceleration of the heartbeat, indicating that considering temporal dynamics in the response of the cardiovascular system is of high importance.

**Funding:** This work was conducted as part of the SleepLoop Flagship of Hochschulmedizin Zürich and funded by the Swiss National Science Foundation (PZ00P3_179795 to CL).

### 2.7. Proof-of-Concept Study Using Phase-Targeted Auditory Stimulation during Sleep to Boost Slow-Wave Activity in Children with Attention-Deficit Hyperactivity Disorder (ADHD)

Elena Krugliakova ^1^, Carina Volk ^1^, Laura Ferster ^2^, Giulia Da Poian ^2^, Walter Karlen^2^ and Reto Huber ^1,3^

^1^ University Children’s Hospital Zürich, Zurich, Switzerland

^2^ Mobile Health Systems Lab., Institute for Robotics and Intelligent Systems, Department of Health Sciences and Technology, ETH Zurich, Zurich, Switzerland

^3^ Department of Child and Adolescent Psychiatry and Psychotherapy, Psychiatric Hospital, University of Zürich, Zürich, Switzerland

**Background**: Slow-wave activity (SWA) during non-rapid eye movement sleep plays an important role in sleep-dependent recovery and attentional performance. In was shown that SWA in children with ADHD might be decreased as compared to their healthy peers (Ringli and Huber, 2011), especially in unmedicated patients (Furrer et al., 2019). To gain insights into the relationship between this SWA decrease and symptoms of ADHD, we employed phase-targeted auditory stimulation (PTAS) as a means to enhance SWA in children with ADHD. **Methods**: We collected sleep hd-EEG data of 18 children (9 children diagnosed with ADHD and 9 control children, both groups 11 ± 1.5 years). Seven children with ADHD received stimulant medication. Two conditions separated by 1 week were carried out: (1) non-stimulation (SHAM) and (2) up-PTAS (STIM) of the slow waves detected over the right prefrontal area. During the stimulation, pink (1/f) noise pulses were delivered in 6 s blocks (ON windows), followed by a 6 s pause (OFF windows). Low SWA (0.5–2 Hz) was assessed separately for STIM ON, STIM OFF, SHAM ON, and SHAM OFF. **Results**: First, we performed mixed ANOVA (within-subject factors: *Condition*, *Window;* between-subject factor: *Group*) on SWA averaged across all electrodes, which showed a significant interaction of the factors *Condition* and *Window* (F(1,16) = 31.37, *p* < 0.001, η^2^ = 0.67). Post-hoc analysis revealed a difference between STIM ON and STIM OFF (t(1,17) = 4.6, *p* < 0.001), and no difference for SHAM ON and SHAM OFF. Topographical analysis showed an increase (*p* < 0.05) in SWA over frontal, vertex, and occipital regions in STIM ON compared to SHAM both for the group of controls and children with ADHD. The SWA increase survived cluster correction only in controls. **Conclusions**: Our results suggest that the PTAS is an efficient tool to boost SWA also in children. Due to the small sample size, no conclusions about group differences can be drawn at this point. We will further investigate how measures of attentional performance relate to the observed effects.

**Funding:** This work was supported by the Borbély-Hess Foundation, Gottfried und Julia Bangerter-Rhyner-Stiftung, Swiss National Science Foundation (grants number 320030_153387 and 320030_179443); the Clinical Research Priority Program (CRPP) “Sleep and Health” of the University of Zurich; and the HMZ Flagship grant “SleepLoop” of the University Medicine Zurich.

### 2.8. Measuring Regulation of Pupil Size under Real-World Conditions

Lazar, R ^1,2,3^ and Spitschan, M ^1,2,4^

^1^ Centre for Chronobiology, Psychiatric Hospital of the University of Basel, Basel, Switzerland

^2^ Transfaculty Research Platform Molecular and Cognitive Neurosciences, University of Basel, Basel, Switzerland

^3^ Department of Psychology, University of Basel, Basel, Switzerland

^4^ Department of Experimental Psychology, University of Oxford, Oxford, UK

**Background**: Pupil size modulates light incident on the retina and is applied as a versatile, non-invasive biomarker for both visual and non-visual processing in humans. Steady-state pupil diameter is largely determined by the activity of the intrinsically photosensitive retinal ganglion cells (ipRGCs) expressing the short-wavelength-sensitive photopigment melanopsin (λmax = 480 nm), which provides a light-sensitive pathway in addition to the canonical photoreceptors—the rods and cones. From tightly controlled laboratory studies employing parametric stimuli, we know that melanopic radiance drives pupil size, with higher melanopic radiance associated with a smaller pupil diameter. However, at present there is no established method or data set assessing variations in pupil size in real-world light exposure. Here, we demonstrate a novel method to assess the light inputs regulating pupil size under dynamic real-world conditions. **Methods**: We integrated a wearable infrared video-based eye tracker (Pupil Labs GmbH) with a small-scale spectroradiometer (Ocean Insight). Both devices were attached to a bespoke, 3D-printed, adjustable head mount and connected to a miniature, battery-driven control computer (Raspberry Pi), enabling simultaneous sampling of pupil size and spectral irradiance in the near-corneal plane at 10 s intervals. We measured natural variation in pupil size across two protocols, in which healthy, young participants (*n* = 7; age range: 20–30 years) moved in and between indoor and outdoor environments varying in light conditions and engaged in a range of everyday tasks. The spectral samples were subjected to a dark and thermal noise calibration using a polynomial noise estimation model, derived from a collected dark spectra database. Melanopic irradiance and photopic lux values were then calculated from the calibrated spectra using the respective standardized CIE spectral sensitivity curves. Absolute pupil diameter was estimated from raw images using a calibrated 3D pupil model. **Results**: We successfully assessed variation in pupil size as a function of near-corneal melanopic irradiance in the real world, yielding distinct dose-response curves for each participant. In line with slow melanopsin signaling, pupil diameter was more accurately predicted by integrating preceding melanopic irradiance values (60 s window) than simultaneous samples. Under these dynamic uncontrolled conditions, data retention was reasonably high (~65% data retained; total number of usable spectrum and pupil size pairs: 6620). The dark and thermal noise calibration reduced deviations in the spectral data to a reasonable level of ≤1 lux. **Conclusions**: In summary, we demonstrated a robust paradigm for ambulatory research on pupil variation in humans outside of the laboratory. In the future, this approach will be used to address real-world pupil assessments in a wide range of research questions in human visual and circadian neuroscience.

**Funding:** R.L. is funded by the European Union’s Horizon 2020 research and innovation programme under the Marie Sklodowska-Curie grant agreement N° 860613. M.S. is supported by a Sir Henry Wellcome Trust Fellowship (Wellcome Trust 204686/Z/16/Z) and a Junior Research Fellowship from Linacre College, University of Oxford. This study was funded by an investigator-initiated research project supported by Ocean Insight.

### 2.9. Are Interictal Epileptiform Discharges during Sleep Causally Related to Slow Waves? Investigating Their Relationship Using Down-Phase-Targeted Auditory Stimulation: A Pilot Study

Sven Leach ^1^, Jelena Skorucak ^1^, Bigna Bölsterli ^2^, Georgia Ramantani ^2^, Bernhard Schmidt ^2^, Giulia Da Poian ^3^, Laura Maria Ferster ^3^, Walter Karlen ^3^ and Reto Huber ^1,4^

^1^ Child Development Center and Pediatric Sleep Disorders Center, University Children’s Hospital Zurich, University of Zurich, Zurich, Switzerland

^2^ Division of Clinical Neurophysiology, University Children’s Hospital Zurich, Zurich, Switzerland

^3^ Mobile Health Systems Lab., Department of Health Sciences and Technology, Institute of Robotics and Intelligent Systems, ETH Zürich, Zurich, Switzerland

^4^ Department of Child and Adolescent Psychiatry and Psychotherapy, Psychiatric Hospital, University of Zurich, Zurich, Switzerland

**Background**: Slow waves (0.5–4 Hz) are the electroencephalographic hallmark of deep sleep. They are associated with interictal epileptiform discharges (IEDs), a pathological sharp waveform in the electroencephalogram typical for patients with epilepsy. However, are slow waves causally related to IEDs? Systematically manipulating slow waves by means of down-phase-targeted auditory stimulation (DPTAS), the presentation of a brief tone time-locked to the down-phase of slow waves, could provide answers. However, its potentially beneficial application in a clinical population—children with epilepsy—was of little avail, possibly due to suboptimal phase precision. The question arises: Can we locally reduce SWA with a phase-optimized stimulation algorithm? **Methods**: In a pilot study, we recorded high-density electroencephalography (HD-EEG) in 14 young healthy adults in a night with and without DPTAS. The stimulation algorithm was based on a phase-locked loop (PLL) with improved phase precision. Auditory stimuli were presented using flat on-ear headphones during stable NREM sleep. More specifically, auditory stimuli were presented during ON windows (16 s), allowing stimulation, which took turns with OFF windows (8 s), withholding stimulation. **Results**: Contrary to what we expected, DPTAS globally increased both low (1–2 Hz) and high SWA (2–4 Hz) during ON windows (time windows with stimulation), both *p* = 0.007; improved the performance in a declarative memory task, *t*(12) = −2.36, *p* = 0.036; and enhanced the capacity to undergo motor learning the next morning, *beta* = −35.75, *t* = −2.49, *p* = 0.024. Interestingly, during OFF windows (time windows without stimulation), DPTAS locally decreased high SWA at a central cluster of 44 electrodes, *p* = 0.026. **Conclusions**: At this point it remains unclear why presenting tones at the down-phase of slow waves resulted in an increase in SWA during time windows with stimulation, yet it challenges the idea that phase precision is the single key factor defining the effect of auditory stimulation on the brain. The observed increase in SWA together with its positive effects on learning and memory is in agreement with the literature. The observed decrease in SWA during OFF windows needs to be further explored.

**Funding:** The project is funded by the Swiss National Science Foundation and the HMZ Flagship Project “SleepLoop” of the University Medicine Zurich Switzerland.

### 2.10. Spectral Slope between 30–45 Hz during NREM and REM Sleep Differentiates between Insomnia Patients and Healthy Controls

Mikutta CA ^1,2^, Feige B ^3^, Desteffani T ^1^, Hertenstein E ^1^, Feher K ^1^, Omlin X ^1^, Schneider C ^1^, Riemann D ^3^ and Nissen Ch ^1^

^1^ University Hospital of Psychiatry and Psychotherapy, University of Bern, Bern, Switzerland

^2^ Privatklinik Meiringen, Meiringen, Switzerland

^3^ Department of Psychiatry and Psychotherapy, Medical Center–University of Freiburg, Faculty of Medicine, University of Freiburg, Freiburg, Germany

**Background:** Sleep in patients with chronic insomnia has been characterized by heightened arousal, reflected by alterations of encephalographic (EEG) oscillatory brain activity. Here, we use the 1/f spectral slope of the electrophysiological power spectrum of 30–45 Hz, which reflects the non-oscillatory, scale-free component of neural activity, as a recent marker of arousal during NREM and REM sleep to further compare sleep in insomnia patients and controls. **Methods:** Data were derived from one sleep laboratory night with 5 channel EEG polysomnographic monitoring in 19 insomnia patients and 19 healthy controls. The differences in the 1/f spectral slope (30–45 Hz) in log–log space during NREM and REM sleep were investigated using multivariate ordinal regression models. **Results:** First, multivariate model analyses in controls showed a significant difference between NREM and REM sleep (F_(1,37)_ = 18.13, *p* < 0.001). Second, multivariate model analyses indicated a significant difference between NREM and REM sleep in controls and insomnia patients (F_(1,36)_ = 4.78, *p* = 0.002), with healthy controls showing lower spectral slope values compared to insomnia patients. **Conclusions:** We demonstrate, to our knowledge the first time, that the spectral slope of the non-oscillatory component differs between insomnia patients and heathy controls. These findings provide further evidence for the hyperarousal model of insomnia.

### 2.11. Circadian VIPergic Neurons of the Suprachiasmatic Nuclei Directly Control Siesta Sleep

Sara Pierre-Ferrer ^1^, Ben Collins ^1^, Christine Muheim ^1,2^, Andrea Spinnler ^1^, Carolina Gutierrez Herrera ^5^, Shao’Ang Wen ^4^, Jochen Winterer ^3^, Mino D. C. Belle ^6^, Hugh D. Piggins ^7^, Michael Hastings ^8^, Andrew Loudon ^9^, Jun Yan ^4^, Csaba Földy ^3^, Antoine Adamantidis ^5,10^ and Steven A. Brown ^1^

^1^ Chronobiology and Sleep Research Group, Institute of Pharmacology and Toxicology, University of Zurich, Winterthurerstrasse 190, 8057 Zurich, Switzerland

^2^ Department of Biomedical Sciences, Washington State University, Spokane, WA 99202, USA

^3^ Laboratory of Neural Connectivity, Brain Research Institute, University of Zurich, Winterthurerstrasse 190, 8057 Zurich, Switzerland

^4^ Institute of Neuroscience, Chinese Academy of Sciences, 320 Yueyang Road, Shanghai 200031, China

^5^ Department of Neurology, Inselspital University Hospital Bern, Freiburgstrasse 18, 3010 Bern, Switzerland

^6^ Institute of Biomedical and Clinical Sciences, University of Exeter Medical School, University of Exeter, Exeter EX4 4PS, UK

^7^ School of Physiology, Pharmacy, and Neuroscience, University of Bristol, Bristol BS8 1TH, UK

^8^ Division of Neurobiology, MRC Laboratory of Molecular Biology, Cambridge CB2 0QH, UK

^9^ Centre for Biological Timing, Faculty of Biology, Medicine and Health, School of Medical Sciences, University of Manchester, Manchester M13 9PT, UK

**Background**: Circadian rhythms in behavior and physiology are controlled and coordinated in mammals by the master clock in the brain, the suprachiasmatic nucleus (SCN) of the hypothalamus. Rodents and humans present similarities in their activity, such as the presence of a midday nap or siesta. It is thought to arise from sleep pressure, but it is not well understood how. Here, we show that siesta sleep is a circadian event controlled by a subpopulation of neurons within the SCN. **Methods**: We looked at electrical activity by staining SCN slices for the immediate–early gene *c-Fos*. Additionally, we recorded electrical firing with a multi-electrode array. To identify which neurons are active at night, we used patch-clamp recordings combined with single-cell sequencing. Finally, optogenetic stimulation and silencing was applied in vivo while recording the EEG signature. **Results**: We demonstrated that a specific population of mouse SCN neurons is active at the “wrong” time of day, at nighttime, when most SCN neurons are silent. Single-cell sequencing allowed us to identify these cells as VIP+ SCN neurons. Furthermore, silencing these active VIP+ neurons resulted in delaying the daily siesta, whereas activating these neurons at another time of the night was sufficient to induce an artificial siesta. **Conclusions**: We were able to show that VIP+ SCN neurons fire at night, at the time of the daily siesta, and that their in vivo electrical manipulation affects siesta sleep. We propose that nighttime siesta in mice is gated by this specific subpopulation of VIPergic neurons in the SCN. Thus, the SCN not only acts as a 24 h metronome, but it can also sculpt features of the sleep–wake cycle.

### 2.12. Does Enhancing Slow Wave Activity Using Auditory Stimulation Boost the Restorative Function of Sleep?

Schneider, N ^1^, Ferster, M L ^2^, Lustenberger, C ^2^, Schlegel, J ^1^, Karlen, W ^2,5^, Huber R ^3,4^, Baumann, C R ^1^ and Maric, A ^1^

^1^ Department of Neurology, University Hospital Zurich, University of Zurich, Zurich, Switzerland

^2^ Department of Health Sciences and Technology, ETH Zurich, Zurich, Switzerland

^3^ Child Development Centre, University Children’s Hospital Zurich, Zurich, Switzerland

^4^ Department of Child and Adolescent Psychiatry, University of Zurich, Zurich, Switzerland

^5^ Institute of Biomedical Engineering, University of Ulm, Ulm, Germany

**Background**: In sleep restriction (SR), the opportunity for sleep-associated restoration is reduced. This leads to increased sleep pressure, reflected by increased initial slow-wave activity (SWA) in the subsequent night. Since slow waves are thought to contribute to restoration, we investigated whether SWA enhancement counteracts this increase. **Methods**: In this study, 14 young male subjects underwent 2 × 7 nights of SR with nightly auditory up-phase stimulation (AS) to enhance SWA in one week (stim) and muted AS in the other week (sham). Sleep was recorded using high-density EEG nets. In a preliminary analysis, we calculated global SWA (spectral power 1–4.5 Hz) in the first 30 min of artefact-free non-REM 2 and 3 sleep in the baseline (BL) night before and the recovery (RC) night after each week of SR. The relative change in SWA between BL and RC was compared across conditions. **Results**: Global SWA change showed a significantly higher increase in the sham (26.66 ± 9.2% SEM) than in the stim condition (1.86 ± 5.1% SEM) across subjects (paired t-test, *p* = 0.048, t = 2.138). **Conclusions**: We found initial indications that AS might boost the restorative function of sleep, reflected in a smaller increase of initial SWA after SR if AS is applied. AS might therefore reduce the effects of insufficient sleep duration.

**Funding:** This project was conducted as part of the Hochschulmedizin Zürich Flagship “SleepLoop.”

### 2.13. Effects of Sleep Deprivation on Task EEG Theta Power

Snipes, S ^1,2^ and Huber, R ^1,3^

^1^ Child Development Centre, University Children’s Hospital Zurich, University of Zurich, Zurich, Switzerland

^2^ Neural Control of Movement Lab, Department of Health Sciences and Technology, ETH Zürich, Zurich, Switzerland

^3^ Department of Child and Adolescent Psychiatry and Psychotherapy, Psychiatric Hospital, University of Zürich, Zurich, Switzerland

**Background**: Sleep deprivation results in an increase in frontal theta power (4–8 Hz oscillations) in the human resting wake EEG (electroencephalogram). Given the association between changes in theta during wakefulness and changes in slow-wave activity (1–4 Hz, i.e., delta) during sleep, it has been hypothesized that theta reflects a form of “local sleep” in wakefulness. However, traditional wakefulness research has separately identified theta as a marker for increased cognitive control during difficult tasks such as arithmetic, short-term memory, and planning, without any relationship to sleep homeostasis. To our knowledge, no study has directly compared task-related theta with sleep-deprivation theta. **Methods**: In an exploratory within-subject experiment, we investigated how theta power changes across the scalp during different tasks and at different levels of sleep pressure. High-density EEG was measured in 18 young healthy adults (9 female, 18–26 years old) performing 6 tasks at 3 levels of sleep pressure. The tasks included a short-term memory task (STM), a lateralized attention task (LAT), a psychomotor vigilance task (PVT), a speech fluency task (SpFT), a game, and music listening. The tasks were performed at baseline in the morning after a normal night of sleep (BL), at the same time in the morning but after only 4 h of sleep (sleep restriction, SR), and after 20 h of additional wakefulness following the short night (sleep deprivation, SD). Task-related theta (trTheta) was evaluated comparing each task at BL with a 7 min rest recording (eyes-open fixation) conducted prior to the task block. Sleep-deprivation theta (sdTheta) was evaluated comparing the tasks from the SD session with the corresponding BL session. **Results**: TrTheta was present in all tasks in frontal-midline and parietal-midline channels, with SpFT resulting in the lowest theta increase relative to rest (Fz hedge’s g = 0.53) and in the fewest channels (19% with *p*-value < 0.05, fdr corrected), whereas the game had the highest theta increase (g = 2.10) and most widespread (69% channels). Theta further increased in all tasks except SpFT following sleep deprivation. SdTheta changes were more widespread in the STM, PVT, and LAT tasks, significantly increasing in 80%, 89%, and 95% of channels, respectively. In the game and music tasks the changes were more localized (33% and 38%, respectively), occurring primarily in frontal-midline channels. **Conclusions**: Our results indicate that changes in theta following sleep deprivation are task dependent, and although the majority of tasks showed an increase, this can occur more or less widespread across the scalp. This may indicate that “local sleep” events in the theta range may occur in addition to underlying task-related theta, and the location of these events further depends on the ongoing task.

**Funding:** No funding.

### 2.14. A Sustained and Vicious Cycle? Bidirectional Relationships between Sleep and Stress in Depressed and Healthy Adolescents

Sarah Schmidt ^1^, Chiara Fontanellaz-Castiglione ^1^, Salome Wild ^1^, Michael Kaess ^1,2^ and Leila Tarokh ^1,3^

^1^ University Hospital of Child and Adolescent Psychiatry and Psychotherapy, University of Bern, Bern, Switzerland

^2^ Section for Translational Psychobiology in Child and Adolescent Psychiatry, Department of Child and Adolescent Psychiatry, Center for Psychosocial Medicine, University Hospital Heidelberg, Heidelberg, Germany

^3^ Translational Research Center, University Hospital of Psychiatry and Psychotherapy, University of Bern, Bern, Switzerland

**Background**: In both adults and adolescents, stress and sleep are closely associated. Existing evidence from animal models suggests that stress exerts a negative influence on sleep, as it is associated with both reduced sleep quality and sleep duration. On the other hand, alterations in sleep behavior can lead to increased feelings of stress. However, the temporal relationships between sleep and stress in adolescence remain understudied in humans. The goal of the current longitudinal study was to investigate the link between sleep and stress in both healthy and depressed adolescents. **Methods**: The current study included 32 medication-free participants, 13 with major depressive disorder (MDD) and 19 healthy controls aged 14 to 17 years (mean = 15.13 (±1.13); 19 girls) recruited as part of a longitudinal study on sleep in adolescent depression. Perceived stress, assessed using the Perceived Stress Scale (PSS), and sleep, measured using the Pittsburgh Sleep Quality Index (PSQI), were measured monthly using a secure online data capture. Outcome variables were the sum score of the PSS and subjective sleep quality and duration derived from the PSQI. On average, 10.41 months of data on sleep quality were available across participants (range = 2 to 15 months). Cross-lagged panel analysis was performed to examine the association between stress and sleep over time. **Results**: Stress and subjective sleep duration were bi-directionally related with higher stress levels in the past month predicting shorter sleep duration in the following month (β −0.04, *p* < 0.001), and conversely, reduced subjective sleep duration was predictive of higher stress (β = −0.06, *p* < 0.01). Stress and subjective sleep quality were unidirectionally related with higher stress scores predicting future lower subjective sleep quality (β = 0.03, *p* < 0.001), whereas lower reported sleep quality was not a significant predictor of more pronounced feelings of stress later on. **Conclusions**: In a sample of adolescents with and without depression, we found bidirectional associations between stress and sleep duration over time. Our findings are in line with previous results in animal models showing a reciprocal relationship between sleep and stress. In addition, our longitudinal study design allows for the elucidation of associations that persists over time. Considering these long-term associations as well as the essential role of sleep for development, our results underline the importance of both stress-reducing and sleep-improving interventions to support mental and physical health in youth.

**Funding:** This research was supported by the Interfaculty Research Cooperation Grant “Decoding Sleep: From Neurons to Health and Mind” from the University of Bern and the Swiss National Science Foundation grant 32003B_184943 (to LT) and the Swiss National Science Foundation grant 32003B_184943 (to L.T.).

### 2.15. Physiological Responsiveness to Phase-Locked Auditory Stimulation during SWS Predicts Increases in Episodic Memory Performance in Older Adults

Wunderlin, M ^1^, Zeller, C ^1^, Nissen, C ^2^, Klöppel, S ^1^ and Züst, M A ^1^

^1^ University Hospital of Old Age Psychiatry and Psychotherapy, University of Bern, Bern, Switzerland

^2^ University Hospital of Psychiatry and Psychotherapy, University of Bern, Bern, Switzerland

**Background**: Previous research suggests that phase-locked acoustic stimulation (PLAS) during SWS is able to boost ongoing oscillatory activity and—as a downstream effect—improve sleep-dependent memory consolidation. Due to an assumed bi-directional link between SWS disturbances and memory decline in aging, older adults might profit most from such interventions. However, current PLAS protocols are arguably optimized for young participants. **Methods**: In this study, 28 participants (age: 61–80 years, M = 69.3; 23 female) were randomly allocated to an intervention or control group. Participants completed one baseline night and three consecutive experimental nights with high-density EEG measurements. The intervention group received PLAS during experimental nights and sham stimulation during the baseline night. The PLAS algorithm was tailored to be less amplitude dependent to better suit older adults’ sleep physiology. In the control group, sham stimulation was applied all four nights. In the evenings before and the mornings after experimental nights, as well as in one-week and three-month follow-up sessions, participants completed a face–occupation-association memory task. **Results**: In the intervention group but not in the control group, PLAS induced an entrained physiological response in all three experimental nights compared to the baseline night. There was no overall difference in memory performance between the intervention and control group. A linear regression model showed that within the intervention group, the physiological response to PLAS predicted memory performance: The higher the amplitude of the entrained slow-wave peak, the better participants’ memory performance starting on the morning of the second experimental night (p_E2_morning_ = 0.012) until the first follow-up (p_E3_evening_ = 0.021, p_E3_morning_ = 0.034, p_FU1_ = 0.027). Responsiveness to stimulation did not correlate with age, education, sleep quality, or other cognitive assessments. **Conclusions**: Using amplitude-less dependent PLAS algorithms as well as multiple nights of intervention might represent necessary steps in the optimization of PLAS protocols for older adults in order for memory effects to unfold.

**Funding:** This work is supported by the Synapsis Foundation, the Peter Bockhoff Foundation, the Heidi Seiler Foundation (2018-PI02), and the Interfaculty Research Cooperation “Decoding sleep” at the University of Bern.

### 2.16. Improving Sleep to Prevent Cognitive Decline

Zeller C ^1^, Wunderlin M ^1^, Klöppel S ^1^, Nissen C^2^ and Züst M A ^1^

^1^ University Hospital of Old Age Psychiatry and Psychotherapy, University of Bern, Bern, Switzerland

^2^ University Hospital of Psychiatry and Psychotherapy, University of Bern, Bern, Switzerland

**Background**: During slow-wave sleep (SWS), the deepest sleep stage, memory is strengthened and metabolic waste-products like amyloid beta are cleared from the brain. With increasing age, sleep naturally becomes more fragmented and disrupted, leading to a loss of SWS. Disrupted sleep has been indicated as an early, modifiable risk factor for cognitive decline. Individuals with mild cognitive impairment and dementia suffer particularly from this fragmentation. In return, impairments of sleep, especially SWS, are likely causally linked to cognitive decline, creating a vicious cycle. We hypothesize that an improvement in SWS would allow the brain to recuperate, potentially decelerating cognitive decline and breaking the vicious cycle. **Methods**: We used auditory closed-loop stimulation to boost slow-wave activity during sleep. **Results**: Preliminary data show that the strength of response as indicated by the amplitude of boosted slow-wave activity predicts increases in memory performance. **Conclusions**: It is important to determine the factors that predict who might profit from this intervention and who might not. To this aim, we devised a two-pronged approach to validate and extend our research protocol. First, to validate our results, healthy participants that had already undergone our intervention were re-tested. This helped identify intra-individual factors that might predict responsiveness to our intervention and gauge potential side effects. Next, we extended our protocol to include individuals with increased risk of developing dementia to test the feasibility of our intervention with individuals who are most in need of it. Our goal was a non-invasive, low-cost, auditory tool to improve SWS and combat cognitive decline.

**Funding:** This work is supported by the Synapsis Foundation grant no. 2018-PI02 (with joint support by the Peter Bockhoff Foundation and the Heidi Seiler Foundation) and the Interfaculty Research Cooperation “Decoding Sleep” at the University of Bern.

## 3. Clinical Science

### 3.1. Sleep Neurophysiology in Unmedicated Adolescents with and without Depression

Fontanellaz-Castiglione C ^1^, Markovic A ^2^, Salvatore V ^1^, Kaess M1 ^3^ and Tarokh L ^1,4^

^1^ University Hospital of Child and Adolescent Psychiatry and Psychotherapy, University of Bern, Bern, Switzerland

^2^ Department of Psychology, University of Fribourg, Fribourg, Switzerland

^3^ Department of Child and Adolescent Psychiatry, Center for Psychosocial Medicine, University Hospital Heidelberg, Heidelberg, Germany

^4^ Translational Research Center, University Hospital of Psychiatry and Psychotherapy, University of Bern, Bern, Switzerland

**Background**: Subjective sleep complaints are often a core symptom of depression and highly prevalent, with 60 to 90% of depressed individuals suffering from disrupted sleep. Studies in adults with depression have shown marked changes in slow-wave activity (SWA); however, findings have been equivocal due to samples with mixed medication status, broad age ranges, and variable severity of depression. In contrast to a considerable literature on adults, few studies have examined sleep neurophysiology in adolescents with and without depression. The aim of the present study was to examine sleep neurophysiology in an unmedicated sample of adolescents with and without major depressive disorder (MDD) using high-density sleep electroencephalogram (EEG). **Methods**: The present study consisted of a sample of 39 adolescents with and without depression between the age of 14 and 17 years of age (mean 15.15 years, SD = 1.1; 25 females; 18 with MDD). Participants were screened for MDD based on a clinical interview. After three adaptation nights where participants followed a sleep schedule ensuring 9 h of sleep per night, high-density sleep EEG (58 EEG derivations) was recorded at participants’ homes. Slow-wave activity (SWA) was calculated as power in the 0.6–4.6 Hz range and an ANOVA with factors of age, sex, and group used to determine statistical differences between the groups. **Results**: We found significantly diminished SWA in adolescents with depression compared to those without. Statistically significant differences were widespread and found in 33 derivations distributed across brain regions. Effect sizes were large, with eta squared values for significant electrodes ranging between 0.11 to 0.28. **Conclusions**: Compared to previous studies, our findings of diminished SWA in adolescents with depression were topographically more widespread and effect sizes were larger. This may have been due to the recruitment of an unmedicated sample, which nonetheless had moderate to severe depression, and the narrow age range may have reduced variability, increasing statistical power. Our findings add to the literature showing impairments in SWA in depression and further our understanding of the role of sleep in depression.

**Funding:** This research was supported by the Interfaculty Research Cooperation grant “Decoding Sleep: From Neurons to Health and Mind” from the University of Bern (to LT) and the Swiss National Science Foundation grant 32003B_184943 (to LT).

### 3.2. More Than Just Legs: Periodic Chin Muscle Activations in a Case of Abdominal RLS

Stephany Fulda, Mauro Manconi and Silvia Riccardi

Sleep Medicine Unit, Neurocenter of Southern Switzerland, Civic Hospital (EOC) of Lugano, Lugano, Switzerland

We present the case of a patient with abdominal restless legs syndrome (RLS), i.e., symptoms fulfilling criteria for RLS but located in the left lower quadrant of the abdomen, who underwent a previous surgical intervention. In repeated nocturnal video-polysomnographies, we observed strikingly rhythmic chin-EMG activations during wakefulness with an inter-movement interval around 24 s, similar to but independent from periodic leg movements, which the patient exhibited during sleep. Both the periodic leg movements and the rhythmic chin activations were responsive to dopaminergic medication.

### 3.3. Cognitive Behavioral Therapy for Insomnia in Patients with Mental Disorders and Comorbid Insomnia: A Systematic Review and Meta-Analysis

Hertenstein E ^1^, Trinca E ^1^, Schneider CL ^1^, Fehér KD ^1^, Su T ^2^, Van Straten A ^3^, Berger T ^4^, Riemann D ^5^, Feige B ^5^ and Nissen C ^1^

^1^ University Hospital of Psychiatry and Psychotherapy, University of Bern, Bern, Switzerland

^2^ Department of Old Age Psychiatry, GGZ inGeest Specialized Mental Health Care, Amsterdam, The Netherlands

^3^ Vrije Universiteit Amsterdam, Faculty of Behavioural and Movement Sciences, Clinical Psychology & Amsterdam Public Health research institute, Amsterdam, The Netherlands

^4^ Department of Clinical Psychology and Psychotherapy, University of Bern, Bern, Switzerland

^5^ Department of Psychiatry and Psychotherapy, Medical Center-University of Freiburg, Faculty of Medicine University of Freiburg, Freiburg, Germany

**Background**: Almost 70% of patients with mental disorders report sleep difficulties and 30% fulfill the criteria for insomnia disorder. Cognitive behavioral therapy for insomnia (CBT-I) is the first-line treatment for insomnia, according to current treatment guidelines. Despite this circumstance, insomnia is frequently treated only pharmacologically, especially in patients with mental disorders. The aim of the present meta-analysis was to quantify the effects of CBT-I in patients with mental disorders and comorbid insomnia on two outcome parameters: the severity of insomnia and the severity of the mental disorder. **Methods**: The databases PubMed, CINHAL (Ebsco), and PsycINFO (Ovid) were searched for randomized controlled trials on adult patients with comorbid insomnia and any mental disorder comparing CBT-I to placebo, waitlist, or treatment as usual using self-rating questionnaires as outcomes for either insomnia or the severity of the mental disorder, or both. **Results**: The search resulted in 1307 records after duplicate removal, of which 22 fulfilled the inclusion criteria and were included for the meta-analysis. The comorbidities were depression (nine studies), post-traumatic stress disorder (PTSD, three studies), depression and PTSD (one study), alcohol dependency (three studies), bipolar disorder (one study), psychosis (one study), and mixed comorbidities (four studies). CBT-I resulted in large effect sizes for the improvement of insomnia and medium effect sizes for the improvement of the severity of mental disorders. **Conclusions**: These significant, stable, medium-to-large effects directly after treatment and at follow-up (3–6 months after end of treatment) indicate that CBT-I is an effective treatment for patients with a mental disorder and comorbid insomnia, especially depression, PTSD, and alcohol dependency, and is also an effective add-on treatment with the aim of reducing the severity of the mental disorder. Thus, in patients with mental disorders and comorbid insomnia, given the many side effects of medication, CBT-I should be considered as a first-line treatment.

### 3.4. Heart Rate Variability in Non-Rapid Eye Movement Sleep Stage 2 Indicates Insomnia and Is Related to Subjective Daytime Performance

Thorsten Mikoteit ^1,2^, Joelle T. Pais Sava ^2^, Marcel A. Zeising ^4^, Edith Holsboer-Trachsler ^1^, Johannes Beck ^1,3^, Serge Brand ^1,5,6,7^ and Martin Hatzinger ^2^

^1^ University of Basel Psychiatric Hospital, Centre for Affective, Stress and Sleep Disorders, Basel, Switzerland

^2^ Psychiatric Services of Solothurn and University of Basel, Solothurn, Switzerland

^3^ Klinik Sonnenhalde, Riehen, Switzerland

^4^ Klinikum Ingolstadt, Centre of Mental Health, Ingolstadt, Germany

^5^ Kermanshah University of Medical Sciences, Kermanshah, Iran

^6^ Department of Sport, Exercise and Health, University of Basel, Basel, Switzerland

^7^ School of Medicine, Tehran University of Medical Sciences, Tehran, Iran

**Objective**: Insomnia disorder is characterized by subjectively perceived poor sleep and impaired daytime performance. However, objective findings of deficits in sleep continuity and cognitive functioning are often mild. The aim of this study was to examine whether objective markers of autonomous hyperarousal, specifically sleep stage-related heart rate variability (HRV), indicate insomnia more reliably than objective sleep continuity measures, and furthermore, whether such biomarkers correlate with poor cognitive daytime performance. **Methods**: Polysomnographic measures of 41 insomniacs (age: 37.9 ± 12.7 years, 56.1% females) were compared to a control group of 27 normal sleepers. Frequency domain measures of HRV (very low- (VLF), low- (LF), and high-frequency (HF) power) were extracted from artefact-free 5 min ECG segments of non-rapid eye movement sleep stage 2 (NREM-S2). Daytime performance was assessed by subjective ratings with insomnia severity index (ISI; items “interference” and “noticeability”) and objective testing of alertness. **Results**: HRV measures in NREM-S2 distinguished between insomnia and normal sleep, with increased NREM-S2-VLF% power (*p* = 0.012, g = 0.702) and decreased NREM-S2-HF% power (*p* = 0.041, g = −0.564) in insomnia. Furthermore, insomniacs presented with both higher perceived impairment of daytime performance (ISI item “noticeability,” *p* < 0.001, g = 0.80) and increased objective reaction time (*p* = 0.084, g = 0.435). Moreover, the above-mentioned NREM-S2-HRV findings in insomnia correlated with both poor subjective daytime performance (“noticeability”; VLF% power: r = 0.334, *p* = 0.013 and HF% power: r = −0.316, *p* = 0.019) and prolonged objective reaction time (VLF% power: r = 0.471, *p* < 0.001 and HF% power: r = −0.348, *p* = 0.008). **Conclusions**: The pattern of HRV findings in NREM-S2 indicates autonomous imbalance and supports the hyperarousal theory of insomnia. In contrast to sleep continuity measures, NREM-S2 HRV reflects the experience of non-restorative sleep and poor cognitive daytime performance more reliably.

**Conflicts of Interest:** The authors declare no conflict of interest.

### 3.5. Sleep Alterations in Unmedicated Patients with Major Depressive Disorder Compared to Healthy Controls

Omlin X ^1^, Fehér K ^1^, Züst M ^2^, Feige B ^2^, Riemann D ^2^ and Nissen C ^1^

^1^ University Hospital of Psychiatry and Psychotherapy, University of Bern, Bern, Switzerland

^2^ Department of Psychiatry and Psychotherapy, Medical Center-University of Freiburg, Faculty of Medicine, University of Freiburg, Freiburg, Germany

**Background**: Sleep disturbances are a common feature in major depressive disorder (MDD). Alterations in REM sleep and decrease in slow-wave sleep have been reported, but findings have varied among studies. However, sleep deprivation (SD) is an effective and rapid-acting treatment in MDD. The synaptic plasticity model proposes that sleep-/wake-dependent shifts in synaptic plasticity represent a critical mechanism of SD in MDD. In light of this model, we re-analyzed baseline PSG data from unmedicated MDD patients and healthy controls in order to assess potential alterations in markers of sleep homeostasis. **Methods**: We compared baseline data from 14 psychiatric inpatients with MDD (age 38.7; 13 m; collected in a sleep phase advance/delay study) and 14 age- and gender-matched healthy controls (age 39.1; 13 m). Patients were free of psychoactive medication for at least 7 days prior to the study (fluoxetine and neuroleptics: 21 days). We analyzed sleep architecture/continuity, EEG spectrum, and slow waves for the first hour of sleep and for the entire night. **Results**: We found a trend towards a reduction in N1 (entire night; *p* = 0.08)), an increase in REM (first hour; *p* = 0.07), and reduced N2 (first hour; *p* = 0.07) for the MDD group. Spectral data from the MDD group showed a significant decrease in alpha power (entire night, *p* = 0.04) and a trend towards less slow and fast spindle power (entire night, *p* = 0.07). No significant differences between groups were found for the remaining sleep variables. **Conclusions**: Data of our MDD group did not show any alterations in markers of sleep homeostasis (e.g., slow-wave sleep/activity/energy or slow-wave variables). The fact that our MDD group was unmedicated, young, and predominantly male might explain these results. Previous studies have shown that factors such as age, gender, and medication status might play an important role in the heterogeneity of findings in MDD.

### 3.6. The Life-ON Study: Sleep and Sleep Disorders during Pregnancy and Postpartum

M Manconi, LC van der Gaag, F Mangili, L Clivio, C Garbazza, S Riccardi, S Mondini, F Furia, E Zambrelli, A D’Agostino, A Cicolin, F Cirignotta and the Life-ON Study Group ^†^

^1^ Fabio Cirignotta MD, University of Bologna, Bologna, Italy

^2^ Susanna Mondini MD, IRCCS Istituto delle Scienze Neurologiche di Bologna, UOC Clinica Neurologica NeuroMet, Bologna, Italy

^3^ Cristina Fonti PhD, IRCCS-Istituto Delle Scienze Neurologiche di Bologna, Bologna, Italy

^4^ Simone Baiardi MD PhD, IRCCS-Istituto Delle Scienze Neurologiche di Bologna, DIMES–University of Bologna, Bologna, Italy

^5^ Rossella Santoro PhD, IRCCS Istituto delle Scienze Neurologiche di Bologna, UOC Clinica Neurologica NeuroMet, Bologna, Italy

^6^ Nicola Rizzo MD, University of Bologna, Bologna, Italy

^7^ Giuliana Simonazzi MD, Division of Obstetrics and Prenatal Medicine, Department of Medical and Surgical Sciences, Sant’Orsola-Malpighi Hospital, University of Bologna, Bologna, Italy

^8^ Alessandra Bianconcini RM, Division of Obstetrics and Prenatal Medicine, Department of Medical and Surgical Sciences, Sant’Orsola-Malpighi Hospital, University of Bologna, Bologna, Italy

**Abstract: Introduction**: Sleep disorders are frequent during pregnancy and puerperium. Yet, objective polysomnographic studies and longitudinal sleep assessments are few and often small in scope. In the Life-ON Study, we conducted the largest polysomnographic study in pregnant women to date and gathered longitudinal subjective assessments of sleep quality. In this paper, we present our findings. **Methods**: Sleep was studied in women participating in the multi-center (Swiss-Italian) Life-ON Study. Women between 18 and 55 years of age and without major morbidities were recruited at a gestational age between 10 to 15 weeks. Information was collected by home polysomnography between the 23rd and 25th week of pregnancy and by sleep-related questionnaires at 11 points in time during pregnancy and one year postpartum. **Results**: The study group included 439 pregnant women (age: mean 33.7; std 4.2) in total, of whom 290 completed the study. Full-night polysomnographic data were available from 353 women. Poor quality of sleep was reported by 34% of women in the first trimester of pregnancy, by 46% of women in the third trimester, and by as many as 71% of women in the first month after delivery. A similar trend was seen for insomnia. Daytime sleepiness peaked in the first trimester in 30% of women and decreased in the third trimester in 22% of women. Prevalence of RLS during pregnancy was 27%, with a peak in the third trimester. Sleep-disordered breathing had a prevalence of 4.2%. A PLMS index larger than 4 was found in 55% of women. A total of 24% of women had a total sleep time of less than 6 h, and 30.6% of women had a sleep efficiency lower than 80%. **Conclusions**: Insomnia, sleepiness, and RLS are highly frequent during pregnancy, with insomnia and daytime sleepiness often persisting also in the first months postpartum. Sleep-disordered breathing was infrequent at the end of the second trimester of pregnancy. A relevant high prevalence of high PLMS scores was recorded, and they had a strong correlation with RLS. 

### 3.7. Obstructive and Central Sleep Apnea in First Ever Ischemic Stroke Are Associated with Different Time Course and Autonomic Activation

Riglietti A ^1^, Fanfulla F ^2^, Pagani M ^3^, Lucini D ^3,4^, Malacarne M ^3,4^, Manconi M ^5,6,7^, Ferretti G ^8,9^, Esposito F ^10,11^, Cereda CW ^12^ and Pons M ^1^

^1^ Department of Pulmonology, Regional Hospital of Lugano (EOC), 6900 Lugano, Switzerland

^2^ Respiratory Function and Sleep Unit–Istituti Clinici Scientifici Maugeri IRCCS, Pavia, Italy

^3^ BIOMETRA Department, University of Milan, Milan, Italy

^4^ Exercise Medicine Unit, Humanitas Clinical and Research Center, Rozzano, Italy

^5^ Sleep and Epilepsy Center, Neurocenter of the Southern Switzerland, Regional Hospital (EOC) of Lugano, Lugano, Switzerland

^6^ Faculty of Biomedical Sciences, Università Della Svizzera Italiana, Lugano, Switzerland

^7^ Department of Neurology, University Hospital, Inselspital, Bern, Switzerland

^8^ Department APSI, University of Geneva, Geneva, Switzerland

^9^ Department of Molecular and Translational Medicine, University of Brescia, Brescia, Italy

^10^ Department of Biomedical Sciences for Health, Università degli Studi di Milano, Milan, Italy

^11^ IRCCS Istituto Ortopedico Galeazzi, Milan, Italy

^12^ Stroke Center EOC, Department of Neurology, Neurocenter of Southern Switzerland Regional Hospital (EOC) of Lugano, Lugano, Switzerland

**Background**: Sleep-related breathing disorders are highly prevalent in patients with ischemic stroke. Among sleep disorder breathing, obstructive sleep apnea is the most represented one, but central sleep apnea, isolated or in the context of periodic breathing/Cheyne–Stokes respiration, is frequently reported in these patients. Altered baroreflex responses were reported in the acute phases of a cerebral event. **Methods**: We conducted, in a group of patients with ischemic stroke (*n* = 60), a prospective 3-month physiological study follow-up to describe the breathing pattern during sleep and baroreflex sensitivity in the acute and in the recovery phase. **Results**: In the acute phase, within 10 days from the onset of symptoms, 22.4% of patients had a normal breathing pattern, 40.3% had an obstructive pattern, 16.4% had a central pattern, and 29.9% showed a mixed pattern. Smaller variations in apnea–hypopnea index were found in the normal breathing and obstructive groups (ΔAHI 2.1 ± 4.1 and −2.8 ± 11.6, respectively) in comparison with the central and mixed pattern groups (ΔAHI −6.9 ± 15.1 and −12.5 ± 13.1, respectively; ANOVA *p* = 0.01). The obstructive group became the most frequent pattern: 38.3% of patients at baseline, 61.7% of patients at follow-up. Modification over time of baroreflex sensitivity was influenced by the site of the lesion and by the sleep disorder pattern in the acute phase (MANOVA *p* = 0.005). **Conclusions**: We suggest a down-regulation of autonomic activity, possibly related to reduced vagal modulation, that may help recovery after stroke or a transitory disconnection with the cortical node that participates in the regulation of sympathetic outflow.

**Funding:** The study was supported by a grant from ABREOC (Scientific Research Advisory Board of the Ente Ospedaliero Cantonale), the Doctor PierLuigi Crivelli Foundation, and the Cecilia Augusta Foundation.

### 3.8. Become Your Own SLEEPexpert: Development and Evaluation of a Web Application to Support a Pragmatic Behavioral Treatment Program for Insomnia in Inpatient Psychiatric Care

Carlotta L. Schneider ^1^, Elisabeth Hertenstein ^1^, Rahel Flückiger ^2^, Kristoffer Fehér ^1^, Franz Moggi ^1^, Thomas Berger ^2^ and Christoph Nissen ^1^

^1^ University Hospital of Psychiatry and Psychotherapy, University of Bern, Bern, Switzerland

^2^ Department of clinical psychology and psychotherapy, University of Bern, Bern, Switzerland

**Background**: Mental disorders are among the leading causes for reduced quality of life due to illness worldwide. The majority of patients with mental disorders suffer from insomnia (disrupted sleep) and insomnia is associated with adverse health outcomes. Current guidelines identify cognitive behavioral therapy for insomnia (CBT-I) as the first-line treatment. However, CBT-I is too complex for patients with severe mental disorders and not systematically implemented in clinical care. Rather, insomnia often remains untreated or treated with hypnotics, related to the risk of adverse effects and dependency. The current project aims to empower patients with mental disorders to take care of their own sleep health based on a behavioral treatment program. **Methods**: We adapted CBT-I in a treatment development phase in collaboration with patients across diagnostic entities (transdiagnostic approach) and health care providers in psychiatric wards (‘*Become your own SLEEPexpert*’). The SLEEPexpert intervention centers on the sleep/circadian science- and evidence-based treatment components of bedtime restriction and circadian adaptation and consists of three phases (therapist-guided treatment initiation, self-management with nurse support, and self-management). **Results**: Pilot data demonstrate feasibility. An improvement in insomnia severity was observed but a control comparison is needed to further test for efficacy. The latest development tests the usability of a web application. Patients receive face-to-face psychoeducation in the ward followed by daily self-management by filling out a web-based form of a sleep diary, including feedback functions, with the support of the nursing team. Pilot data show heightened interest from patients and health care providers and a willingness to use a web-based form of the SLEEPexpert program. A smartphone application is currently being developed with the aim to improve attractiveness and usability. **Conclusions**: The project is expected to result in a novel sleep-centered intervention that has the potential to be implemented and disseminated in routine clinical care for patients with severe mental disorders. Given the substantive burden of insomnia and mental disorders, the proposed developments are expected to be of high public health relevance.

### 3.9. Restless Legs Syndrome and Polysomnographic Features in Patients with Multiple Sclerosis

Davide Sparasci ^1^, Raffaele Ferri ^2^, Anna Castelnovo ^1,3^, Silvia Miano ^1^, Kosuke Tanioka ^4^, Naoko Tachibana ^5^, Chiara Carelli ^1^, Gianna Riccitelli ^6^, Giulio Disanto ^6^, Chiara Zecca ^3,6^, Claudio Gobbi ^3,6^ and Mauro Manconi ^1,3^

^1^ Sleep Medicine Unit, Neurocenter of Southern Switzerland, Ospedale Civico, Lugano, Switzerland

^2^ Sleep Research Centre, Department of Neurology I.C., Oasi Institute for Research on Mental Retardation and Brain Aging (IRCCS), Troina, Italy.

^3^ Faculty of Biomedical Sciences, Università Della Svizzera Italiana, Lugano, Switzerland

^4^ Department of Somnology, Tokyo Medical University, Tokyo, Japan

^5^ Division of Sleep Medicine, Kansai Electric Power Medical Research Institute, Osaka, Japan

^6^ Multiple Sclerosis Center, Neurocenter of Southern Switzerland, Ospedale Civico, Lugano, Switzerland

**Background**: The goal was to estimate the prevalence of restless legs syndrome (RLS) and periodic limb movements during sleep (PLMS), and related features such as fatigue, drowsiness, and depression, in patients with multiple sclerosis (MS). **Methods**: A cross-sectional, observational, controlled, polysomnographic investigation was conducted. Eighty-six patients with a diagnosis of MS underwent a telephone interview assessing the five standard diagnostic criteria for RLS. Patients also filled in questionnaires for fatigue, depression, sleepiness, subjective sleep quality, and quality of life. Seventy-six participants underwent polysomnography (PSG) and a maintenance of wakefulness test (MWT). Twenty-eight healthy controls and 35 patients with idiopathic RLS (iRLS) were recruited to compare sleep architecture and sleep-related leg movement activity (LMA) with patients affected by MS. **Results**: MS patients without RLS presented increased sleep latency, percentage of sleep stage N1, and reduced total sleep time compared to healthy controls. The prevalence of RLS and PLMS (PLMSI ≥ 15/h) in patients with MS was 31.4% and 31.6%, respectively. Among MS patients with RLS, only 37.5% had a PLMSI ≥ 15/h (71.4% in iRLS). PLMS in patients with RLS and MS were fewer and shorter, but with similar periodicity when compared to iRLS. RLS and PLMS were independently correlated to fatigue. PLMS without RLS was associated with higher wakefulness after sleep onset and stage shifts per hour, increased stage N1, and a reduction in stage N3. When assessed by MWT, somnolence and fatigue did not overlap each other. **Conclusions**: MS is a risk factor for RLS, PLMS, and lower sleep quality in comparison to healthy patients. The low percentage of patients with RLS with a high PLMSI, together with the absence of correlation between RLS and female gender and older age, support the existence of a distinct symptomatic form of RLS in MS. Our results suggest a dissociation between motor (PLMS) and sensory symptoms (RLS sensory component) in RLS secondary to MS, with possible treatment implications. Finally, particular attention should be given to symptoms of RLS in fatigued MS patients.

### 3.10. Cognitive Behavioral Therapy for Insomnia in Patients with a Psychiatric Comorbidity: A Systematic Review and Meta-Analysis Focusing on Comorbid Symptoms

Hertenstein E ^1^, Trinca E ^1^, Wunderlin M ^2^, Schneider C L ^1^, Züst M A ^2^, Fehér K D ^1^, Su T ^3^, Van Straten A ^4^, Berger T B ^5^, Riemann D ^6^, Feige, B ^6^ and Nissen, C ^1^

^1^ University Hospital of Psychiatry and Psychotherapy, University of Bern, Bern, Switzerland

^2^ University Hospital of Old Age Psychiatry and Psychotherapy, University of Bern, Bern, Switzerland

^3^ Department of Old Age Psychiatry, GGZ inGeest Specialized Mental Health Care, Amsterdam, The Netherlands

^4^ Vrije Universiteit Amsterdam, Faculty of Behavioural and Movement Sciences, Clinical Psychology& Amsterdam Public Health research institute, Amsterdam, The Netherlands

^5^ Department of Clinical Psychology and Psychotherapy, University of Bern, Bern, Switzerland

^6^ Department of Psychiatry and Psychotherapy, Medical Center-University of Freiburg, Faculty of Medicine University of Freiburg, Freiburg, Germany

**Background**: According to current guidelines, cognitive behavioral therapy for insomnia (CBT-I) is the gold standard for the treatment of insomnia. A third of patients with mental disorders fulfil the criteria for insomnia disorder. Hence, the aim of this meta-analysis was not only to quantify the effect of CBT-I in patients with mental disorders and comorbid insomnia on the insomnia severity, but also on the severity of the comorbid mental disorder. **Methods**: On the basis of a systematic literature search on the databases PubMed, CINHAL (Ebsco), and PsycINFO (Ovid), eligible studies were identified. The aim was to find randomized controlled trials reporting original data on the effect of CBT-I on insomnia severity and the severity of the mental disorders. Studies with control groups, allowing for a conclusion on the efficacy of the study intervention such as waitlist, placebo, or treatment as usual, were included. Furthermore, the diagnosis of the insomnia disorder and the mental comorbidity had to be based on the *Diagnostic and Statistical Manual of Mental Disorders* (DSM) or the *International Statistical Classification of Diseases and Related Health Problems* (ICD). **Results**: The comorbidities were depression (nine studies), post-traumatic stress disorder (PTSD, three studies), alcohol dependency (three studies), bipolar disorder (one study), psychosis (one study) and mixed comorbidities (five studies). Regarding the severity of the comorbid mental disorder, CBT-I had a medium effect immediately after treatment and small effect at follow-up. **Conclusions**: CBT-I is an effective treatment for patients with mental disorders and comorbid insomnia. Not only insomnia severity, but also the severity of the mental disorders was reduced after treatment. In conclusion, psychiatric hospitals should revert to CBT-I to treat insomnia in inpatients with mental disorders. However, CBT-I should only be regarded as an add-on to the treatment as usual for mental disorders. More randomized controlled trials investigating the effect on different mental disorders are needed. Meaningful long-term effect studies should be conducted.

**Funding:** The publication of these proceedings received no external funding.

**Conflicts of Interest:** The authors declare no conflict of interest. 

